# Understanding Comorbidities and Their Contribution to Predictors of Medical Resource Utilization for an Age- and Sex-Matched Patient Population Living With HIV: Cross-Sectional Study

**DOI:** 10.2196/13865

**Published:** 2019-09-11

**Authors:** Michelle Odlum, Sunmoo Yoon

**Affiliations:** 1 Columbia University School of Nursing New York, NY United States; 2 Columbia University Irving Medical Center New York, NY United States

**Keywords:** HIV, sex differences, Charlson scores, comorbidity, electronic health records, health resource

## Abstract

**Background:**

More than 60% of people aging with HIV are observed to have multiple comorbidities, which are attributed to a variety of factors (eg, biological and environmental), with sex differences observed. However, understanding these differences and their contribution to medical resource utilization remains challenging as studies conducted exclusively and predominantly among males do not translate well to females, resulting in inconsistent findings across study cohorts and limiting our knowledge of sex-specific comorbidities.

**Objective:**

The objective of the study was to provide further insight into aging-related comorbidities, their associated sex-based differences, and their contribution to medical resource utilization, through the analysis of HIV patient data matched by sex.

**Methods:**

International Classification of Disease 9/10 diagnostic codes that comprise the electronic health records of males (N=229) and females (N=229) were categorized by individual characteristics, chronic and mental health conditions, treatment, high-risk behaviors, and infections and the codes were used as predictors of medical resource utilization represented by Charlson comorbidity scores.

**Results:**

Significant contributors to high Charlson scores in males were age (beta=2.37; 95% CI 1.45-3.29), longer hospital stay (beta=.046; 95% CI 0.009-0.083), malnutrition (beta=2.96; 95% CI 1.72-4.20), kidney failure (beta=2.23; 95% CI 0.934-3.52), chemotherapy (beta=3.58; 95% CI 2.16-5.002), history of tobacco use (beta=1.40; 95% CI 0.200-2.61), and hepatitis C (beta=1.49; 95% CI 0.181-2.79). Significant contributors to high Charlson scores in females were age (beta=1.37; 95% CI 0.361-2.38), longer hospital stay (beta=.042; 95% CI 0.005-0.078), heart failure (beta=2.41; 95% CI 0.833-3.98), chemotherapy (beta=3.48; 95% CI 1.626-5.33), and substance abuse beta=1.94; 95% CI 0.180, 3.702).

**Conclusions:**

Our findings identified sex-based differences in medical resource utilization. These include kidney failure for men and heart failure for women. Increased prevalence of comorbidities in people living long with HIV has the potential to overburden global health systems. The development of narrower HIV phenotypes and aging-related comorbidity phenotypes with greater clinical validity will support intervention efficacy.

## Introduction

### Background

A variety of comorbidities characterize long-term survivorship with HIV, which is not merely explained by the decrease in AIDS-related mortality. An average of 3 aging-related comorbidities are observed in 60% to 90% of people living with HIV/AIDS (PLWH), aged 50 years and above [1,2]. They are attributed to antiretroviral toxicity, persistent immunodeficiency, and inflammation [2]. Biological sex-related differences also contribute to the determinants of such aging-related comorbidities in populations living with HIV [3,4]. Sex differences are observed in the pathogenesis of HIV and other infectious diseases. Differences exist between males and females for a variety of factors including biological, genetic, environmental, and sociobehavioral [3,4]. Studies have also found sex differences in HIV viral and immunological response as well as disease progression [3,5]. However, challenges exist in clinical studies to isolate biological sex differences and gain a more in-depth understanding of how HIV affects health-related outcomes. Current gaps in knowledge include the lack of understanding of the impact of sex-based differences on the presence of non-AIDS-related comorbidities [4,5]. The presence of multiple comorbidities, observed in HIV, include characteristics of aging-associated phenotypes such as disability and frailty [6]. However, phenotypes are studied far more in the aging field, which is not specific to HIV [7-9]. Poor health outcomes such as disability and frailty increase the risk of poor functional status, which complicates access to care and disrupts disease self-management, resulting in increased medical resource utilization [9]. Therefore, effective interventions require the identification of narrower phenotypes with greater clinical validity [7,10,11]. Exploratory studies should report outcomes based on sex to determine when such differences warrant more focused investigations [4]. HIV research that defines sex differences will ensure intervention efficacy in males and females and will allow for the observation of pathway differences to support effective HIV treatment and ultimately cure.

### Objectives

This study provides an understanding of sex-related differences and has identified multifactorial determinants of aging-related comorbidities and their contributions to medical resource utilization, which are represented by Charlson comorbidity scores [12-14]. This cross-sectional study looked at the presence of comorbidities and not HIV-related contributions (eg, disease stage and immune status) to the development or proliferation of comorbidities. Clinical data that comprise electronic health records (EHRs) were analyzed, and within- and between-group differences identified for a patient population of PLWH were matched by sex. An exploration of HIV clinical data is required to gain further insights into the sex-based differences that may have clinical consequences and contribute to increased medical resource utilization. In fact, higher Charlson scores indicate the increased likelihood that a predicted outcome will result in higher resource utilization or 1-year mortality [12,15,16]. As this study focused on morbidity, not mortality, Charlson scores served as an indicator of medical resource utilization. Here, we report the following: (1) the prevalence of comorbidities by sex and (2) predictors of medical resource utilization represented by Charlson comorbidity scores that comprise the factors of individual characteristics, chronic conditions, mental health conditions, treatment, high-risk behaviors, and infections by sex. Our findings can inform the design and implementation of effective interventions to reduce the chronic disease burden, decrease medical resource utilization, and support successful aging with HIV [3,4].

## Methods

### Patient Population

We analyzed EHR data for HIV-infected males (N=229) and females (N=229) matched on sex. Records were retrieved from a New York City clinical data warehouse for adult inpatients aged 18 years and older from January 2006 to December 2014. Institutional review board approval was obtained from the Columbia University Irving Medical Center to analyze the deidentified EHRs data, which excluded all potentially identifiable patient information (eg, name, address, and date of birth). Patients were not involved in data analysis or interpretation. Patient personal contact information was not shared with investigators. After data cleaning, which included the removal of incomplete International Classification of Disease (ICD) 9/10 codes, a total of 786 HIV-infected males (N=524) and females (N=262) remained for matching, identified as HIV infected by HIV-related diagnostic codes. Mahalanobis propensity score matching was used to find the female patients comparable to male patients [17]. Female patients were matched to male patients with the closest propensity scores. After the removal of unmatched data, a sample of 229 males and 229 females remained. Propensity score matching allows for meaningful comparisons between groups and reduces confounding factors in the statistical assessment of outcomes [17]. We developed a dataset very limited in missing data, as clinical datasets are known to have a variety of missing data elements. Diagnostic codes (ICD 9/10) came from past medical histories, clinical encounters, and problem lists. Diagnostic codes were organized under the factors of individual characteristics (eg, ICD9/10: 262—malnutrition), chronic conditions (eg, ICD9/10: 401, 401.1, 401.9—hypertension), mental health conditions (eg, ICD9/10: 311—depressive disorders), treatment (eg, ICD9/10: V58.11—chemotherapy), high-risk behaviors (eg, ICD9/10: 305.1—tobacco use), and infections (eg, ICD9/10: 70.54—hepatitis B). Patients were distributed into nonmutually exclusive groups based on sex. Inclusion in a diagnostic group was the existence of the diagnostic code in the patient chart history.

### Statistical Analysis

We examined the predictors of medical resource utilization represented by Charlson comorbidity scores. We summarized our results with descriptive statistics, bivariate analyses, and linear regression models. To determine the relationship between identified predictors and Charlson scores, we calculated Pearson Product Moment Correlations. *t* tests assessed the differences in continuous variables, and chi-square test assessed the differences in categorical variables. A total of 3 independent stepwise multiple regressions (ie, all patients, male only, and female only) were performed to identify the relative importance of significant Charlson score predictors (*P*<.05). A stepwise approach was used to prevent bias in the selection of variables in the final models [18]. We report betas and CIs for regression analyses. SPSS 23.0 was used to conduct data analysis.

## Results

### Patient Characteristics

We included 458 patients in our analysis, aged 18 to 85 years, with the mean age of 50.3 (SD 14.1 years). The racial distribution of the female sample (N=229) includes 39.7% (91/229) blacks, 14.4% (33/229) whites, 0.4% (1/229) Asian, 0.4% (1/229) Native American, 0.8% (2/229) Native Hawaiian/Pacific Islanders, 11.9% (27/229) other, and 32.5% (74/229) unknown or declined. The male sample (N=229) includes 23.3% (53/229) blacks, 26.5% (61/229) whites, 0.8% (2/229) Asian, 0.4% (1/229) Native American, 0.4% (1/229) Native Hawaiian/Pacific Islanders, 13.9% (32/229) other, and 34.6% (79/229) unknown or declined. The average length of hospital stay was 9.65 (SD 11 days) for males and 9.45 (SD 13 days) for females. For males, the average HIV RNA viral load distribution was ≤500 copies/mL: 24.1% (55/229); 500-4999 copies/mL: 41.4% (95/229); 5000-49,999 copies/mL: 20.7% (47/229); and ≥50,000 copies/mL: 13.8% (32/229). The average CD4+ lymphocyte counts (CD4) for males were ≤200 cells/µL: 16.5% (38/229); 201-349 cells/µL: 22.7% (52/229); 350-500 cells/µL: 24.4% (56/229); and ≥501 cells/µL: 36.4% (83/229), with 63.6% (146/229) prescribed antiretrovirals. For females, the average viral load distribution was ≤500 copies/mL: 31.5% (71/229); 500-4999 copies/mL: 27.8% (64/229); 5000-49,999 copies/mL: 27.8% (64/229); and ≥50,000 copies/mL: 13.1% (30/229). The average CD4 counts for females were ≤200 cells/µL: 15.4% (35/229); 201-349 cells/µL: 17.9% (41/229); 350-500 cells/µL: 21.4% (49/229); and ≥501 cells/µL: 45.3% (104/229), with 72% (165/229) prescribed antiretrovirals. The top 6 ICD9/10 codes for males were hypertension, current tobacco use, noncompliance with treatment/regimen (not following the treatment or regimen prescribed for improved health outcomes) [19], hyperlipidemia, history of tobacco use, and depression. The top 6 ICD9/10 codes for females were hypertension, current tobacco use, and history of tobacco use, uncomplicated asthma, acute kidney failure, and hyperlipidemia (Table 1). Charlson scores ranged from 0 to 20, with an average of 7.72 (SD 9.7) for males, and 0 to 18, with an average of 6.90 (SD 9.45) for females (Tables 1 and 2).

**Table 1:** Descriptive statistics for medical resource utilization (Charlson scores).

Variable		Males (n=229)	Females (n=229)	All (N=458)
**Individual characteristics**				
	Age, mean (SD)	51.5 (13)	51.5 (13)	50.3 (14.1)
	Length of hospital stay, mean (SD)	9.65 (11)	9.45 (13)	9.58 (12)
	Malnutrition, n (%)	35 (15.2)	25 (10.9)	60 (13.1)
	Noncompliance with treatment/regimen, n (%)	38 (16.5)	32 (13.9)	70 (15.2)
**Chronic conditions, n (%)**				
	Diabetes mellitus II	25 (10.9)	29 (12.6)	54 (11.7)
	Hyperlipidemia	37 (16.5)	35 (15.2)	72 (15.7)
	Hypertension	55 (24.0)	62 (27.0)	117 (25.5)
	Atherosclerosis	31 (13.5)	18 (7.8)	49 (10.6)
	Atrial fibrillation	11 (4.8)	5 (2.1)	16 (3.4)
	Heart failure	23 (10.0)	24 (10.4)	47 (10.2)
	Uncomplicated asthma	19 (8.2)	37 (16.5)	56 (12.2)
	Acute kidney failure	30 (13.1)	37 (16.5)	67 (14.6)
**Mental health conditions**				
	Depressive disorder	35 (15.2)	31 (13.5)	66 (14.4)
**Treatment**				
	Chemotherapy	25 (10.9)	18 (7.8)	43 (9.3)
**High-risk behaviors**				
	Substance abuse	33 (14.4)	19 (8.2)	52 (11.3)
	Current tobacco use	54 (23.5)	40 (17.4)	94 (20.5)
	History of tobacco use	36 (15.7)	39 (17.0)	75 (16.3)
**Infections**				
	Chronic hepatitis C	33 (14.4)	27 (11.7)	60 (13.1)

**Table 2:** Correlations and *P* values for medical resource utilization (Charlson scores).

Variable		Males (n=229)		Females (n=229)		All (N=458)	
		Correlation	*P* value	Correlation	*P* value	Correlation	*P* value
**Individual characteristics**							
	Age	0.359	.001^a^	0.118	.14	0.24	.001^a^
	Length of hospital stay	0.186	.001^a^	0.148	.05^b^	0.169	.02^b^
	Malnutrition	0.289	.001^a^	0.085	.26	0.198	.18
	Noncompliance with treatment/regimen	−0.01	.90	0.058	.38	0.024	.66
**Chronic conditions**							
	Diabetes mellitus II	0.026	.64	−0.054	.51	−0.016	.74
	Hyperlipidemia	0.053	.49	0.082	.20	0.068	.31
	Hypertension	0.084	.22	−0.058	.53	0.011	.17
	Atherosclerosis	0.242	.001^a^	−0.008	.73	0.138	.001^a^
	Atrial fibrillation	0.101	.14	−0.005	.21	0.061	.28
	Heart failure	0.153	.03^b^	0.159	.04^b^	0.155	.04^b^
	Uncomplicated asthma	−0.024	.70	0.026	.60	−0.003	.96
	Acute kidney failure	0.195	.001^a^	0.12	.10	0.153	.03^b^
**Mental health conditions**							
	Depressive disorder	−0.028	.64	−0.036	.62	−0.03	.45
**Treatment**							
	Chemotherapy	0.197	.001^a^	0.167	.05^b^	0.185	.001^a^
**High-risk behaviors**							
	Substance abuse	−0.002	.94	0.151	.05^b^	0.069	.42
	Current tobacco use	−0.042	.49	−0.09	.25	−0.06	.31
	History of tobacco use	0.183	.001^a^	0.139	.04^b^	0.159	.001^a^
**Infections**							
	Chronic hepatitis C	0.175	.001^a^	0.107	.14	0.144	.001^a^

^a^*P*<.01.

^b^*P*<.05.

### Predictors of Medical Resource Utilization

Bivariate analyses revealed significant contributions to high Charlson scores for a variety of factors in our patient population. These include the individual characteristics of older age (≥50 years; *X*^2^_19_=149.9), longer length of hospital stay (*t*_456_=2.96), and noncompliance with treatment/regimen (*X*^2^_19_=30.8). In addition to the chronic conditions of hyperlipidemia (*X*^2^_19_=31.2), hypertension (*X*^2^_19_=54.5), atherosclerosis (*X*^2^_19_=38.6), acute kidney failure (*X*^2^_19_=32.7), and heart failure (*X*^2^_19_=53.5), significant differences also included the treatment of chemotherapy (*X*^2^_19_=81.8), the high-risk behavior of current tobacco use (*X*^2^_19_=31.6) and history of tobacco use (*X*^2^_19_=35.9), and the infection of chronic hepatitis C (*X*^2^_19_=54.8; Table 3).

Bivariate analyses also revealed significant contributions to high Charlson scores within sex groups for a variety of factors. For males, these include the individual characteristics of older age (≥50 years; *X*^2^_19_=80.5), longer length of hospital stay (*t*_227_=2.60), and malnutrition (*X*^2^_19_=19.7), in addition to the chronic conditions of atherosclerosis (*X*^2^_19_=44.4), acute kidney failure (*X*^2^_19_=32.3), and heart failure (*X*^2^_19_=51.5). Significant differences also included the treatment of chemotherapy (*X*^2^_19_=55.4), the high-risk behavior of history of tobacco use (*X*^2^_19_=41.3), and the infection of chronic hepatitis C (*X*^2^_19_=38.1; Table 3). For females, these include the individual characteristic of older age (≥50 years; *X*^2^_19_=77.8) in addition to the chronic conditions of hyperlipidemia (*X*^2^_19_=31.2) and hypertension (*X*^2^_19_=52.3). Significant differences also included the treatment of chemotherapy (*X*^2^_19_=32.3), the high-risk behavior of substance abuse (*X*^2^_19_=36.8), and the infection of chronic hepatitis C (*X*^2^_19_=30.4; Table 3).

The stepwise multiple regression for all patients identified the most significant (*P*<.05) contributors to high Charlson scores to be the individual characteristics of older age (beta=1.91; 95% CI 1.22-2.60), longer length of hospital stay (beta=.039; 95% CI 0.12-0.065), and malnutrition (beta=1.88; 95% CI 0.882-2.87); chronic conditions of acute kidney failure (beta=1.29; 95% CI 0.347-2.24) and heart failure (beta=1.22; 95% CI 0.104-2.33); treatment of chemotherapy (beta=3.37; 95% CI 2.22-4.53); history of high-risk behavior of tobacco use (beta=1.03; 95% CI 0.130-1.93); and infection of chronic hepatitis C (beta=1.10; 95% CI 0.097-2.109; Table 4).

**Table 3:** Chi-square/*t* tests for outcome variable: medical resource utilization (Charlson scores).

Variable		Males (n=229)		Females (n=229)		All (N=458)	
		*X*^2^ (*df*)	*P* value	*X*^2^ (*df*)	*P* value	*X*^2^ (*df*)	*P* value
**Individual characteristics**							
	Age <50 years and ≥50 years	80.5 (19)	.001^a^	77.8 (19)	.001^a^	49.9 (19)	.001^a^
	Length of hospital stay^b^	−2.60 (227)	.001^a^	−1.18 (227)	.07	−2.96 (456)	.001^a^
	Malnutrition	42.8 (19)	.001^a^	17.3 (19)	.15	26.9 (19)	.10
	Noncompliance with treatment/regimen	19.7 (19)	.26	20.5 (19)	.12	30.8 (19)	.04^c^
**Chronic conditions**							
	Diabetes mellitus II	23.7 (19)	.13	13.8 (19)	.16	14.3 (19)	.15
	Hyperlipidemia	18.8 (19)	.15	31.2 (19)	.03^c^	31.2 (19)	.03^c^
	Hypertension	28.2 (19)	.07	52.3 (19)	.001^a^	54.5 (19)	.001^a^
	Atherosclerosis	44.4 (19)	.001^a^	9.8 (19)	.26	38.6 (19)	.001^a^
	Atrial fibrillation	29.1 (19)	.06	10.4 (19)	.22	21.5 (19)	.11
	Acute kidney failure	32.3 (19)	.03^c^	27.1 (19)	.08	32.7 (19)	.03^c^
	Heart failure	51.5 (19)	.001^a^	22.8 (19)	.14	53.5 (19)	.001^a^
	Uncomplicated asthma	16.5 (19)	.17	22.5 (19)	.13	16.3 (19)	.15
**Mental health conditions**							
	Depressive disorder	30.4 (19)	.06	11.9 (19)	.15	19.0 (19)	.12
**Treatment**							
	Chemotherapy	55.4 (19)	.001^a^	32.3 (19)	.03^c^	81.8 (19)	.001^a^
**High-risk behaviors**							
	Substance abuse	21.2 (19)	.14	36.8 (19)	.001^a^	28.0 (19)	.07
	Current tobacco use	29.1 (19)	.07	13.1 (19)	.22	31.6 (19)	.03^c^
	History of tobacco use	41.3 (19)	.001^a^	19.2 (19)	.13	35.9 (19)	.01^c^
**Infections**							
	Chronic hepatitis C	38.1 (19)	.001^a^	30.4 (19)	.01^c^	54.8 (19)	.001^a^

^a^*P*<.01.

^b^Length of hospital stay values display *t* tests for outcome variable: medical resource utilization.

^c^*P*<.05.

**Table 4:** Linear regression models of best fit for medical resource utilization (Charlson Scores).

Variables			Unstandardized coefficient beta, mean increases (95% CI for beta)	*P* value
**All patients model, N=458**				
	**Individual characteristics**			
		Age <50 years and ≥50 years	1.91 (1.218-2.595)	.001^a^
		Length of hospital stay	.039 (0.012-0.065)	.004^a^
		Malnutrition	1.88 (0.882-2.873)	.001^a^
	**Chronic conditions**			
		Acute kidney failure	1.29 (0.347-2.241)	.008^a^
		Heart failure	1.22 (0.104-2.328)	.03^b^
	**Treatment**			
		Chemotherapy	3.37 (2.218-4.529)	.001^a^
	**High-risk behaviors**			
		History of tobacco use	1.03 (0.130-1.932)	.03^b^
	**Infections**			
		Chronic hepatitis C	1.10 (0.097-2.109)	.03^b^
**Male-only model, N=229**				
	**Individual characteristics**			
		Age <50 years and ≥50 years	2.37 (1.446-3.286)	.001^a^
		Length of hospital stay	.046 (0.009-0.083)	.02^b^
		Malnutrition	2.96 (1.721-4.204)	.001^a^
	**Chronic conditions**			
		Acute kidney failure	2.23 (0.934-3.521)	.001^a^
	**Treatment**			
		Chemotherapy	3.58 (2.164-5.002)	.001^a^
	**High-risk behaviors**			
		History of tobacco use	1.40 (0.200-2.607)	.02^b^
	**Infections**			
		Chronic hepatitis C	1.49 (0.181-2.791)	.03^b^
**Female-only model, N=229**				
	**Individual characteristics**			
		Age <50 years and ≥50 years	1.37 (0.361-2.375)	.008^a^
		Length of hospital stay	.042 (0.005-0.078)	.03^b^
	**Chronic conditions**			
		Heart failure	2.41 (0.833-3.984)	.003^a^
	**Treatment**			
		Chemotherapy	3.48 (1.626-5.328)	.001^a^
	**High-risk behaviors**			
		Substance abuse	1.94 (0.180-3.702)	.03^b^

^a^*P*<.01.

^b^*P*<.05.

The final stepwise multiple regression model for male patients identified the most significant (*P*<.05) contributors to high Charlson scores to be the individual characteristics of older age (beta=2.37; 95% CI 1.45-3.29), longer length of hospital stay (beta=.046; 95% CI 0.009-0.083), and malnutrition (beta=2.96; 95% CI 1.72-4.20) in addition to the chronic conditions of acute kidney failure (beta=2.23; 95% CI 0.934-3.52); the treatment of chemotherapy (beta=3.58; 95% CI 2.16-5.002), the history of high-risk behavior of tobacco use (beta=1.40; 95% CI 0.200 to 2.61), and the infection of chronic hepatitis C (beta=1.49; 95% CI 0.181-2.79), as the most significant (*P*<.05) contributors to high Charlson scores (Table 4).

The final stepwise multiple regression model for female patients identified the most significant (*P*<.05) contributors to high Charlson scores to be the individual characteristics of older age (beta=1.37; 95% CI 0.361-2.38) and longer length of hospital stay (beta=.042; 95% CI 0.005-0.078) in addition to the chronic conditions of heart failure (beta=2.41; 95% CI 0.833-3.98); the treatment of chemotherapy (beta=3.48; 95% CI 1.626-45.33), the high-risk behavior of substance abuse (beta=1.94; 95% CI 0.180-3.402), as the most significant (*P*<.05) contributors to high Charlson scores (Table 4).

## Discussion

### Principal Findings

With immune restoration and viral suppression leading to long-term survivorship with HIV, there is a need to increase our focus on the management and prevention of comorbidities. Therefore, it is essential to improve our understanding of aging-related comorbidities and sex differences in HIV clinical outcomes and survival. To further explore these differences and their contribution to medical resource utilization, we analyzed EHR data for HIV-infected patients matched by sex.

Phenotype frameworks view aging within a broader context of objectively defined phenotypic manifestations (eg, comorbidities and physical-social functioning). We observed the sex-related interplay between individual characteristics, chronic conditions, treatment, mental health conditions, high-risk behaviors, and infections and their contribution to medical resource utilization.

Our results contribute to the development of narrower HIV phenotypes with greater clinical validity. Charlson comorbidity scores are robust predictors of both medical resource utilization and 1-year mortality [15,16,20] and are essential for epidemiological investigations on age and survival [18]. However, the use of Charlson scores to understand medical resource utilization in populations of PLWH with comorbidities remains sparse [14,21]. Although no significant differences existed in mean Charlson scores for males and females, contributions to medical resource utilization differed based on sex.

The lack of significant differences in Charlson scores for our matched sample is not reflected in the published literature as studies have shown that males have significantly higher morbidity rates than females [4,22]. However, the contribution of factors to the utilization of medical resources was different for males and females [12]. Our regression models for our patient population identified age as a significant contributor to high Charlson scores. Age was the second highest for all patients, first in the male-only model and third in the female-only model. Similar to aging in uninfected populations, males exhibited higher Charlson scores based on age [23]. Results were different for females as the treatment of chemotherapy was the most significant contributor to high Charlson scores, followed by heart failure. Chemotherapy was the second most significant contributor in males, and heart failure was not in the male-only model. Antineoplastic chemotherapy is understandably a significant contributor to medical resource utilization as such treatment is a consequence of diagnosed malignancies [24,25]. Their toxic effects can result in inadequate nutrition, making patients vulnerable to malnourished states [26]. Although malnourishment was not significant in the female-only model, it was the third significant contributor in the all patient and male-only models. Non-AIDS-defining cancers are increasing in populations of PLWH [27], which includes coinfection by oncogenic viruses such as hepatitis C virus (HCV), which is also a significant contributor in our all patient and male-only models. Heightened cancer risk includes behavioral risk factors as well, such as cigarette smoking. History of tobacco use was also a significant contributor in our all patient and male-only models. Antineoplastic agents are a significant problem in populations of PLWH, with potentially overlapping toxicities with antiretroviral therapy [24,25]. A better understanding of such interactions will be critical for cancer survival in this population.

Heart failure was the second most significant contributor in the female-only model, eighth in the all patient model and not present in the male-only model. Cardiovascular-related illness is a known risk factor in PLWH, with heart disease being a common complication [28,29]. PLWH have increased cardiovascular disease–related mortality compared with uninfected groups [5,27]. A longitudinal study revealed that risk of cardiovascular-related mortality increased steadily for PLWH from 1999 to 2013, with a decrease in risk observed in uninfected groups [30,31]. Similar to our findings, previous studies have indicated an increased risk of myocardial infarction and stroke in females compared with males [4,5]. Females have higher inpatient mortality after myocardial infarction at younger ages than males, with greater complications after invasive interventions. Cardiovascular-related outcomes for females living with HIV compared with males also include more severe strokes, longer length of hospital stay, and higher mortality [5,27]. Length of hospital stay was also a significant contributor in all 3 models and a major financial burden on the US health care system. HIV-related hospitalizations are characterized by some of the more expensive diagnostic categories [3,18]. In populations of PLWH with no comorbidities, studies have shown a 60% increase in length of stay and a 70% increase in medical resource utilization, compared with uninfected populations [32]. Our heart failure results align with the literature on HIV-infected females. However, studies have shown lower uptake of cardiovascular disease–related interventions among females with HIV compared with males [3].

Acute kidney failure was the fourth most significant contributor in the male-only model, fifth in the all patient model, and not present in the female-only model. Although HIV nephropathy has decreased with antiretroviral therapy, compared with uninfected groups, the prevalence of kidney disease remains high for PLWH of all ages [27]. Studies have shown that kidney-related comorbidities are associated with extremely high medical costs, as indicated in our models [27]. Kidney disease is a major burden to HIV as a result of risk factors including HCV coinfection [29,33]. HCV was a significant contributor in the male-only and all patient models, aligning with our kidney disease findings and consistent with the natural history of HCV among PLWH [28,29]. The amplified effects of HCV on HIV cellular replication are known to go beyond liver-related diseases and increase the likelihood of inflammation-related illnesses such as non-AIDS cancers and cardiovascular diseases in this population as well [30,33,34]. Given the prevalence of HCV in PLWH, studies are needed to model ways in which HCV accounts for the development of comorbidities to support primary prevention [30,34].

Although research has shown higher substance use in males living with HIV compared with females, substance abuse was reported in the female-only model. Moreover, 8% of females living with HIV report substance abuse, with significantly higher reports in populations of males living with HIV, particularly men who have sex with men [18]. In our dataset, substance abuse is a combined variable comprising substance use disorders, including alcohol, cannabis, stimulants, hallucinogens, and opioids [22,31]. A substance use diagnosis puts patients at great risk for developing comorbidities, resulting in its significant contribution to high Charlson scores. Diagnoses of opioid abuse have resulted in the exacerbation of existing comorbidities and make medical and treatment adherence difficult [31]. Five times higher viral loads are seen in PLWH who report the use of stimulants [31,35], which is a consequence of poor adherence to antiretroviral use, increased utilization of emergency health care resource, and increased rates of chronic conditions [31,35].

### Limitations

The paper is a cross-sectional analysis of diagnostic codes from EHRs. We did not assess multiple comorbidities in our cross-sectional study, and trends in comorbidities over time were not evaluated. We analyzed documented diagnoses of comorbidities for people living with HIV. We do not explore HIV-related contributions (eg, disease stage and immune status) to the development or severity of comorbidities, only their documented presence or absence. Therefore, diagnosis dates were not considered. Future longitudinal studies in similar populations should account for these additional factors, track HIV disease stage and immune status over time, and utilize different analytical approaches to explore the development of comorbidities and their contributions to medical resource utilization. Our sample was not matched on other factors such as socioeconomic status. EHR data are collected during the course of clinical care and not collected for research purposes. Understandably, sociodemographic information was incomplete for a variety of indicators including race. We did not explore the contribution of HIV-related clinical indicators (CD4, viral load, and antiretrovirals). As a cross-sectional study, the analysis of HIV-related clinical indicators one point in time would not be informative to the presence and absence of the observed comorbidities. Future longitudinal studies should analyze HIV-related clinical indicators over time to explore their potential contribution to the development of comorbidities.

Certain antiretroviral medications are linked to increased cardiovascular risk [30,31]. Therefore, antiretroviral regimens and changes in regimens over time should be included in future analytic studies as well.

### Conclusions

Our analysis provides evidence to further support insight into long-term survivorship with HIV. Similarities and differences were observed between HIV-infected males and females and factor-specific contributions to medical resource utilization. Our findings contribute to the literature on sex-based differences with HIV infection and aging-related comorbidities or phenotype development in aging populations of PLWH. Moreover, cohort studies report that females are better controllers of HIV, although the mechanisms have been unclear [9,11,36]. Sex-specific mechanisms of protection must also be explored in future studies as females generally demonstrate phenotypes of viral control [37]. Targeted interventions should also include nonclinical differences between males and females, such as health education for effective symptom management. In a study that comprises HIV-infected males and females with similar demographics and clinical characteristics, more females acknowledged asking clinicians about the symptoms of aging-related comorbidities and were provided such information without request. The study also revealed that 80% of females desired symptom-related information compared with 22% of males [36,38]. To date, little attention is given to interventions targeting HIV aging phenotypes and sex-based differences, with HIV-infected populations generally being ignored in intervention research [36-38]. Biological sex must be considered in clinical intervention development and implementation to improve HIV- and health-related outcomes for males and females.

## Abbreviations

EHR: electronic health record
HCV: hepatitis C virus
ICD: International Classification of Disease
PLWH: people living with HIV/AIDS
